# *Plasmodium vivax* Malaria in Pregnant Women in the Brazilian Amazon and the Risk Factors Associated with Prematurity and Low Birth Weight: A Descriptive Study

**DOI:** 10.1371/journal.pone.0144399

**Published:** 2015-12-16

**Authors:** Camila Bôtto-Menezes, Mônica Caroline Silva dos Santos, Janicéia Lopes Simplício, Jandira Menezes de Medeiros, Kelly Cristina Barroso Gomes, Isabel Cristina de Carvalho Costa, Eva Batista-Silva, Cristiana Teixeira do Nascimento, Eda Cristina da Silva Chagas, José Felipe Jardim Sardinha, Franklin Simões de Santana Filho, Marianna Brock, Azucena Bardají, Flor Ernestina Martínez-Espinosa

**Affiliations:** 1 Programa de Pós-Graduação em Medicina Tropical, Universidade do Estado do Amazonas, Manaus, Amazonas, Brazil; 2 Universidade Federal do Amazonas, Manaus, Amazonas, Brazil; 3 Fundação de Medicina Tropical Dr. Heitor Vieira Dourado (FMT-HVD), Manaus, Amazonas, Brazil; 4 ISGlobal, Barcelona Ctr. Int. Health Res. (CRESIB), Hospital Clínic-Universitat de Barcelona, Barcelona, Spain; 5 Centro de Pesquisas Leônidas e Maria Deane/Fiocruz Amazônia, Manaus, Amazonas, Brazil; Ehime University, JAPAN

## Abstract

**Introduction:**

*Plasmodium vivax* is the most prevalent malaria species in the American region. Brazil accounts for the higher number of the malaria cases reported in pregnant women in the Americas. This study aims to describe the characteristics of pregnant women with malaria in an endemic area of the Brazilian Amazon and the risk factors associated with prematurity and low birth weight (LBW).

**Methods/Principal Findings:**

Between December 2005 and March 2008, 503 pregnant women with malaria that attended a tertiary health centre were enrolled and followed up until delivery and reported a total of 1016 malaria episodes. More than half of study women (54%) were between 20–29 years old, and almost a third were adolescents. The prevalence of anaemia at enrolment was 59%. Most women (286/503) reported more than one malaria episode and most malaria episodes (84.5%, 846/1001) were due to *P*. *vivax* infection. Among women with only *P*. *vivax* malaria, the risk of preterm birth and low birth weight decreased in multigravidae (OR, 0.36 [95% CI, 0.16–0.82]; p = 0.015 and OR 0.24 [95% CI, 0.10–0.58]; p = 0.001, respectively). The risk of preterm birth decreased with higher maternal age (OR 0.43 [95% CI, 0.19–0.95]; p = 0.037) and among those women who reported higher antenatal care (ANC) attendance (OR, 0.32 [95% CI, 0.15–0.70]; p = 0.005).

**Conclusion:**

This study shows that *P*. *vivax* is the prevailing species among pregnant women with malaria in the region and shows that *vivax* clinical malaria may represent harmful consequences for the health of the mother and their offsprings particularly on specific groups such as adolescents, primigravidae and those women with lower ANC attendance.

## Introduction

In 2011 the region of the Americas reported nearly half million malaria cases, 77% of them were due to *Plasmodium vivax*. According to the World Health Organization, the American region was responsible in the same year for nearly 20% of the total *P*. *vivax* malaria cases reported in the world [1].

In 2007 it was estimated that 92.9 million pregnancies occurred in *P*. *vivax* endemic areas and of them that 2.9 million pregnancies (3.1%) occurred in the American region [2]. In 2008 a total of 5,740 malaria cases were reported in pregnant women in the Americas, but it is estimated that the number of malaria cases in pregnant women was much higher. A 75% of the reported cases in the region were from Brazil (4315/5740) [3] and of the latter, 571 cases were reported only in the Manaus city area, representing 13.2% of Brazil cases and 10% of the Americas [4].

It is well known that pregnant women have a higher risk for *P*. *falciparum* infection and disease, and the impact of *P*. *falciparum* infection on pregnancy outcomes, such as maternal anaemia, low birth weight (LBW) and prematurity are well documented, at least in sub-Saharan Africa [5, 6]. There is evidence that *P*. *vivax* infection can be associated with low birth and anaemia, as it has been described in Thailand, India and Papua (Indonesia) [7–12]. In the region of the Americas, several studies have described that *P*. *vivax* infection during pregnancy is associated with maternal anaemia, low birth weight, preterm delivery and miscarriage [13–18].

The current study aims to characterize malaria among pregnant women from an endemic area of the Brazilian Amazon, and to assess in this population group the risk factors associated with poor pregnancy outcomes.

## Methods

### Ethical aspects

The study protocol was approved by the Research Ethics Committee of the Fundação de Medicina Tropical Dr. Heitor Vieira Dourado (FMT-HVD) (study registration No. 0836-05/2005) and the same committee approved an amendment to the protocol in 2010 (study registration No. 2013). We obtained informed written consent from all participants and from the guardians on behalf of the pregnant women less than 18 years old.

### Study area

The city of Manaus (3° 1'S, 60° 02'O) is located in the Brazilian Amazon region, capital of the Amazonas state, northern Brazil. Manaus is one of the top six cities contributing to the Gross National Product, which together represent approximately 25% of the Brazilian Gross National Product. The population of the city of Manaus was of 1,802.525 inhabitants according to the 2010 census. It is the most populous city in the northern region and the seventh in Brazil. The majority of the population (99.5%) lives in urban areas [19]. Approximately 38,500 live births are recorded every year in Manaus, and most births (99%) are attended by skilled health personnel in health facilities and 80% of pregnant women report at least four ANC [20]. The Annual Parasitic Index in Manaus was of 39.2/1,000 inhabitants in 2005 and 14.6/1,000 inhabitants in 2008, being classified as a medium risk malaria transmission area (10 to 49.9 cases/1.000 inhabitants) [21].

The Brazilian National Malaria Control Program relies on early diagnosis and treatment of malaria cases in general population [22]. Since 2006 the Ministry of Health recommends active detection of malaria infection with microscopy at each ANC visit to all pregnant women living in the Amazon region [23]. In Brazil, malaria is a notifiable disease and every confirmed case has to be reported through a national surveillance system (SIVEP) that includes information on pregnancy status [4]. All malaria cases are laboratory-confirmed by light microscopy. Malaria treatment is given for free and provided exclusively through the public health system. Standard treatment for non-severe *P*. *vivax* malaria in pregnant women consists of oral chloroquine (1500 mg, over three days) followed by weekly prophylaxis with chloroquine (300 mg, over 12 weeks). During the study period weekly prophylaxis recommendation changed from getting started after the second *P*. *vivax* malaria episode during pregnancy to start after the first one. The treatment is completed with primaquine six months after delivery [24]. The chloroquine resistance in the area is 10% in the general population [25]. There is no data available about therapeutic failure or frequency of relapses among pregnant women in the Manaus area [26].

### Study population and study design

From December 2005 to March 2008, pregnant women of any gestational age attending to the outpatient clinic of the FMT-HVD, and diagnosed with malaria due to any *Plasmodium* species confirmed by microscopy, were invited to participate in the study. The FMT-HVD is a tertiary health care centre that runs a passive case detection system (outpatient clinic) for pregnant woman among other curative services. After written informed consent was given, blood samples were collected for parasitaemia determination and anaemia screening. According to Brazilian national guidelines, all confirmed malaria cases received treatment, and clinical follow up was done on days (D) 3, 7, 14, 21, 28 and 35 after treatment initiation. After D35, follow up visits were scheduled every four weeks until delivery. In the event of a new malaria episode the woman initiated the same sequence of follow up visits again. All pregnant women with anaemia received ferrous sulphate and folic acid according to the Brazilian national guidelines.

Information on the number of antenatal visits, date of birth, birth weight, gender of the newborn, and the occurrence of any congenital malformation was retrieved through the Live Birth Information System (SINASC) [27]. Birth weight was measured on a digital scale at the health facility level by nurses within the first hour after birth. Those women who had miscarriage, stillbirth or multiple gestations (twins or triplets) as a pregnancy outcome were excluded from the analysis. The gestational age was assessed by ultrasound, or in the absence of this, by the date of the last menstrual period. The SINASC database from 2005 to 2008 for the Amazonas state was provided by the Foundation for Health Surveillance of the Amazon (FVS). All other variables were obtained at the time of each scheduled visits by study staff.

### Definitions

A malaria case was defined as the presence of asexual forms of any *Plasmodium* species in peripheral blood of any density by microscopy in pregnant women with symptoms. For the purpose of the analysis, the duration of any single malaria episode was estimated as 28 days, in order to distinguish between the same episode and a new one. Low birth weight was defined as a birth weight less than 2,500 g. Low birth weight was classified, in turn, as very low birth weight (between 1,000 and 1,499 g), and as extreme low birth weight (less than 1,000 g). For the analysis of birth weight in relation to gestational age, Brazilian standardized growth curves [28] were used to avoid any deviations from the Lubchenco growth reference curves that are based on Caucasian populations in industrialized countries [29]. Sex specific growth curves were calculated and compared, given the gender specific differences in foetal growth described in the literature [30]. Newborns were classified as large for gestational age when the estimate was higher than the 90^th^ percentile, appropriate for gestational age when it was between the 10^th^ and the 90^th^ percentiles, and small for gestational age when it was below the 10^th^ percentile [28]. The intra-uterine growth retardation (IUGR) was defined as the newborn small for gestational age. Anaemia during pregnancy was defined as a haemoglobin level less than 11 mg/dL. Mild, moderate and severe anaemia were defined as a haemoglobin levels of 9–10.9 mg/dL, 7–8.9 mg/dL, and less than 7 mg/dL, respectively. Prematurity was defined as a gestational age less than 37 weeks.

### Statistical analysis

All clinical and laboratory data from patients were collected through standardized questionnaires and recorded in Epi Info software (version 6.04, CDC, Georgia, USA). Analysis was performed using STATA software (version 12, College Station, Texas, USA). The analysis was stratified in two groups: 356 women with only *P*. *vivax* during pregnancy and 503 women with any *Plasmodium* species during pregnancy. The comparison between the proportions in the group with and without prematurity and LBW, respectively, was validated by the chi-square statistical test. To calculate the odds ratio (OR) logistic regression analysis was used. To evaluate the association between maternal factors, and prematurity and LBW multivariate analysis was performed using backward stepwise logistic regression, removing variables with a p value of 0.05. All episodes occurred during pregnancy were included and were further categorised in single episode, two episodes or at least 3 episodes during pregnancy.

## Results

**Fig 1:**
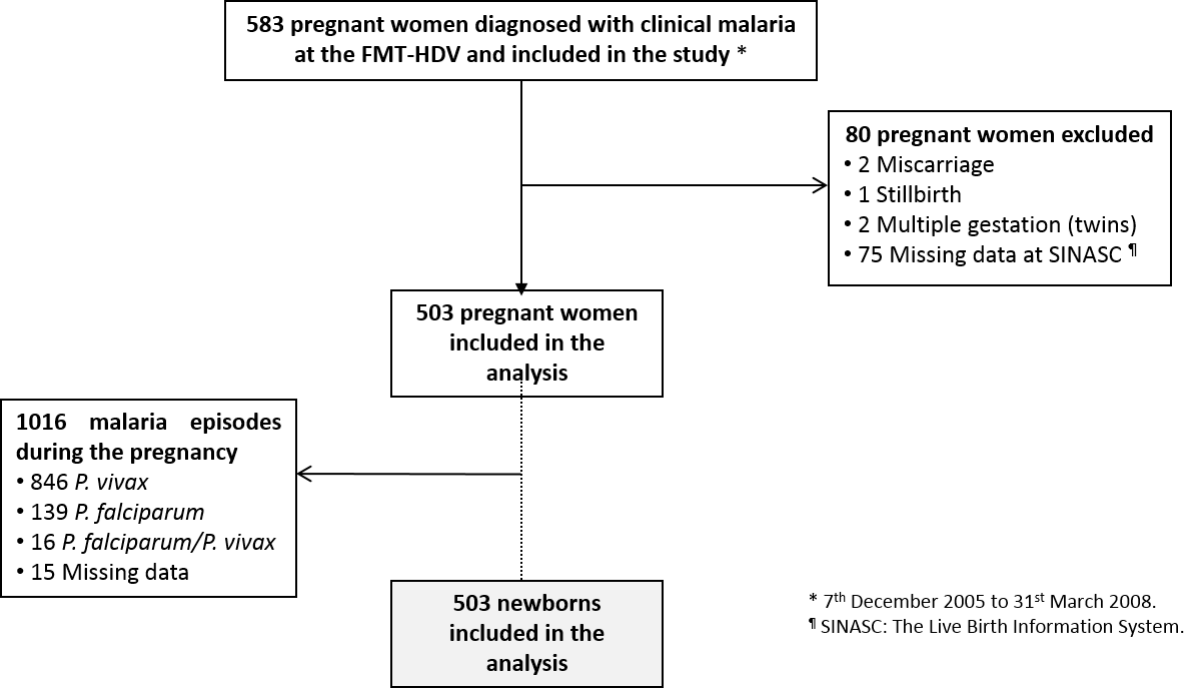
Study profile.

Between December 2005 and March 2008, a total of 583 pregnant women were enrolled in the study at the FMT-HVD, of which 503 were finally included in the analysis (Fig 1).

### Characteristics of the study pregnant women and their newborns

See Table 1 for baseline characteristics of study women. More than half of study women (54%, 274/502) were between 20 and 29 years old, and almost a third were adolescents. Most women were in the second (170/440) or third trimester (213/440) at enrolment (39% and 48%, respectively) and thirteen percent of study pregnant women were in their first trimester. Most women had at least primary studies (68%, 323/475) and were residents in the Manaus area (72%, 353/488). More than hundred women (130/488) came from other cities in the Amazonas region, three women came from another Brazil state (Roraima and Rondônia states) and two women came from border nations (Republic of Guyana and French Guyana). Most of them had at least four ANC visits (73%, 354/486). The prevalence of anaemia at enrolment was 59% (265/449; haemoglobin mean ± SD of 10.6 ± 1.5), of which 77% (204/265) was mild anaemia and 1.5% severe anaemia.

**Table 1:** Baseline characteristics of study pregnant women.

Characteristics		n (%) or Mean ± SD^a^
Age at enrolment (years) (n = 502)		23.7 ± 6.1
Age at enrolment (n = 502)	9 to 19	141 (28)
	20 to 29	274 (54)
	30 to 39	79 (16)
	≥ 40	8 (2)
Gravidity (n = 489)	Primigravidae	150 (31)
	2 to 3 pregnancies	205 (42)
	≥ 4 pregnancies	134 (27)
Gestational age at enrolment (weeks) (n = 440)		23.8 ± 8.9
Gestational age at enrolment (n = 440)^b^	1st trimester	57 (13)
	2^nd^ trimester	170 (39)
	3^rd^ trimester	213 (48)
Level of education (n = 475)	Illiterate	4 (1)
	Primary school	323 (68)
	Secondary school	137 (29)
	Higher education	11 (2)
Place of residence (n = 488)	Manaus area	353 (72)
	Outside Manaus area	135 (28)
Total number of antenatal visits(throughout pregnancy) (n = 486)	None	26 (5)
	1 to 3	106 (22)
	4 to 6	208 (43)
	≥ 7	146 (30)
Clinical malaria episodes during pregnancy (any *Plasmodium* species) (n = 503)	1	217 (43.2)
	2	146 (29)
	≥ 3	140 (27.8)
Haemoglobin level at enrolment (mg/dL) (n = 449)		10.6 ± 1.5
Anemia at enrolment (n = 449)	Yes	265 (59)
Categorization for anemia at enrolment (n = 265) ^c^	Mild anemia	204 (77)
	Moderate anemia	57 (21.5)
	Severe anemia	4 (1.5)

^a^Standard deviation.

^b^1st trimester: 0–12 weeks, 2nd: 13–24 weeks, 3rd: 25–40 weeks.

^c^Mild anemia: haemoglobin levels of 9–10.9 mg/dL, moderate: 7–8.9 mg/dL, severe: < 7 mg/dL.

**Fig 2:**
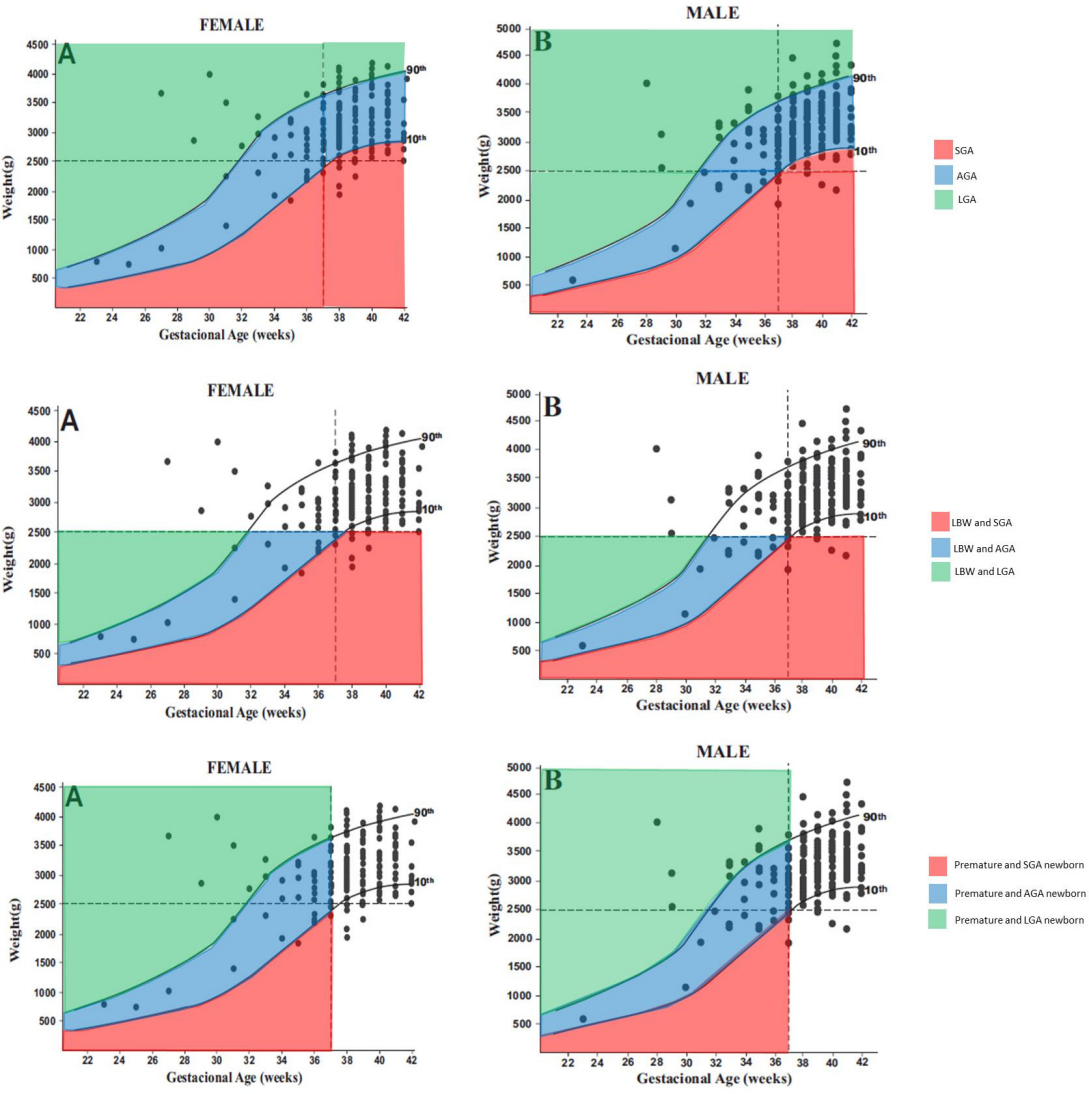
Correlation between gestational age and birth weight and graphical representation of the 10th and 90th percentiles for females (A) and males (B) based on Brazilian-specific growth curves. SGA: Small for gestational age (< 10th percentile); AGA: Appropriate for gestational age (10th-90th percentile); LGA: Large for gestational age (>90th percentile); LBW: Low birth weight (<2500 g); Premature newborn (< 37 weeks). The intra-uterine growth retardation (IUGR) was defined as the newborn SGA. No differences were found by gender to IUGR (p = 0.686), LBW (p = 0.406) and prematurity (p = 0.968).

Table 2 summarizes newborns characteristics. The prevalence of preterm birth was 14.8% (65/438). The prevalence of low birth weight was 9% (45/503), of which four cases were very low birth weight, and four cases were extreme low birth weight. Most newborns (78.4%, 333/425) had a birth weight appropriate for gestational age, 12.7% (54/425) were large for gestational age, and 8.9% (38/425) were small for gestational age (IUGR); no differences were found by gender among babies with IUGR (8.1% in male and 9.7% in female newborns, p = 0.686) (see Fig 2). The inter-rater agreement between the study cohort whose birth weight was retrieved from the SINASC system and a sub-sample of newborns (n = 98) whose birth weight was collected from the newborn card by study staff was assessed using the Kappa statistic, showing a high correlation agreement [Kappa score 0.951 (95% CI 0.855 to 1.000)].

**Table 2:** Baseline characteristics of newborns of study pregnant women.

Characteristics		n (%) or Mean ± SD^a^
Gender (n = 501)	Male	252 (50.5)
	Female	249 (49.5)
Birth weight (g) (n = 503)		3142 ± 558
Low birth weight (<2500 g) (n = 503)	Yes	45 (9)
Categorization for low birth weight (n = 45)	Low birth weight	37 (82)
	Very low birth weight	4 (9)
	Extreme low birth weight	4 (9)
Premature delivery (< 37 weeks) (n = 439)	Yes	65 (14.8)
Classification by birth weight and gestational age (n = 425)^b^	Large for gestational age (LGA)	54 (12.7)
	Appropriate for gestational age (AGA)	333 (78.4)
	Small for gestational age (SGA)^c^	38 (8.9)
Congenital malformation (n = 454)^d^	Yes	2 (0.4)

^a^Standard deviation.

^b^LGA >90th percentile; AGA: 10th-90th percentile; SGA < 10th percentile [28].

^c^The intra-uterine growth retardation (IUGR) was defined as the newborn small for gestational age.

^d^Determination by clinical examination.

### Frequency of *Plasmodium* species during pregnancy

*Plasmodium vivax* infection frequency in the first malaria episode during the pregnancy was 80.5% (395/491), followed by *Plasmodium falciparum* (18.1%, 89/491), and mixed *P*. *falciparum* and *P*. *vivax* infection (1.4%, 7/491). Most women (56.9%, 286/503) reported two or more malaria episodes during the same pregnancy. The 503 study women reported a total of 1016 malaria episodes (ranging from one to eight episodes per woman). Most episodes were due to *P*. *vivax* (84.5%, 846/1001), followed by *P*. *falciparum* (13.9%, 139/1001), and mixed *P*. *falciparum* and *P*. *vivax* infection (1.6%, 16/1001). Most women (72.1%, 356/494) had only malaria due to *P*. *vivax* during pregnancy. The frequency distribution of *P*. *vivax* in the first malaria episode during pregnancy among pregnant women increased over the study period from 76.8% (199/259) in the first half of the study to 84.5% (196/232) in the second half of the study. The frequency of *P*. *falciparum* decreased from 20.9% (54/259) in the first half of the study to 15.1% (35/232) in the second half over the same period.

### Risk factors for prematurity

Table 3 shows the analysis of maternal factors and their association with the risk of preterm birth. Among women with only *P*. *vivax* malaria during pregnancy, the risk of premature delivery decreased with higher maternal age at enrolment (OR for women 20 or more years old, 0.43 [95% CI, 0.19–0.95]; p = 0.037), higher parity (OR for two or more pregnancies, 0.36 [95% CI, 0.16–0.82]; p = 0.015) and in those women with higher attendance to ANC (OR for four or more ANC visits, 0.32 [95% CI, 0.15–0.70]; p = 0.005). Maternal anaemia at enrolment was significantly associated with an increased risk of premature delivery (OR, 2.35 [95% CI, 1.11–5.01]; p = 0.026) in the univariate analysis but this was not confirmed in the adjusted analysis.

**Table 3:** Risk factors for premature delivery among study pregnant women with malaria.

Maternal risk factors		Only *P*. *vivax* during pregnancy (n = 356)						any *Plasmodium* species (n = 503)					
		Univariate model			Multivariate model			Univariate model			Multivariate model		
		OR^a^	(95% CI)^b^	*P* value^c^	OR^a^	(95% CI)^b^	*P* value^c^	OR^a^	(95% CI)^b^	*P* value^c^	OR^a^	(95% CI)^b^	*P* value^c^
Age at enrolment (years)	< 20	1	…	…	1	…	…	1	…	…	1	…	…
	≥ 20	0.33	(0.17–0.63)	**0.001**	0.43	(0.19–0.95)	**0.037**	0.43	(0.25–0.72)	**0.001**	0.54	(0.29–0.99)	**0.047**
Gravidity	Primigravidae	1	…	…	1	…	…	1	…	…	1	…	…
	≥ 2 pregnancies	0.42	(0.22–0.79)	**0.007**	0.36	(0.16–0.82)	**0.015**	0.55	(0.32–0.93)	**0.026**	0.59	(0.32–1.12)	0.106
Gestational age at enrolment	1st trimester	1	…	…				1	…	…			
	2^nd^/3^rd^ trimester	1.31	(0.49–3.54)	0.589				1.44	(0.63–3.32)	0.390			
Level of education (n = 475)	Illiterate/ Primary school	1	…	…				1	…	…			
	Secondary school/ Higher education	0.78	(0.41–1.51)	0.467				0.82	(0.47–1.43)	0.481			
Place of residence (n = 488)	Manaus area	1	…	…				1	…	…			
	Outside Manaus area	0.74	(0.35–1.57)	0.433				0.99	(0.57–1.74)	0.985			
Total number of antenatal visits(throughout pregnancy)	< 4	1	…	…	1	…	…	1	…	…	1	…	…
	≥ 4	0.45	(0.23–0.87)	**0.018**	0.32	(0.15–0.70)	**0.005**	0.50	(0.29–0.86)	**0.013**	0.49	(0.28–0.87)	**0.014**
Clinical malaria episodes during pregnancy (any *Plasmodium* species)	1	1	…	…	1	…	…	1	…	…	1	…	…
	≥ 2	0.68	(0.37–1.27)	0.226	0.50	0.25–1.03)	0.060	0.66	(0.39–1.09)	0.106	0.68	(0.40–1.17)	0.164
*Plasmodium* species at first episode in the pregnancy	*P*. *vivax*	…	…	…				1	…	…			
	*P*. *falciparum*	…	…	…				1.29	(0.68–2.43)	0.419			
	*P*. *falciparum/vivax*	…	…	…				2.16	(0.41–11.44)	0.364			** **
Anemia at enrolment	No anemia	1	…	…	1	…	…	1	…	…			
	Overall anemia	2.35	(1.11–5.01)	**0.026**	2.09	(0.94–4.65)	0.072	1.62	(0.92–2.86)	0.097			

^a^Odds Ratio.

^b^95% confidence interval.

^c^Chi-squared test.

The reduced risk of preterm birth in women of higher maternal age and greater attendance to ANC was also seen among women with malaria due to any *Plasmodium* species (OR for women 20 or more years old, 0.54 [95% CI, 0.29–0.99]; p = 0.047, and OR for having reported four or more ANC visits, 0.49 [95% CI, 0.28–0.87]; p = 0.014) (see Table 3).

### Risk factors for low birth weight

Table 4 shows the analysis of maternal factors and their association with LBW. In women with only *P*. *vivax* malaria during pregnancy, the risk of LBW decreased with higher parity (OR for mothers with two or more pregnancies, 0.24 [95% CI, 0.10–0.58]; p = 0.001).

**Table 4:** Risk factors for low birth weight among study pregnant women with malaria.

Maternal risk factors		Only *P*. *vivax* during pregnancy (n = 356)						any *Plasmodium* species (n = 503)					
		Univariate model			Multivariate model			Univariate model			Multivariate model		
		OR^a^	(95% CI)^b^	*P* value^c^	OR^a^	(95% CI)^b^	*P* value^c^	OR^a^	(95% CI)^b^	*P* value^c^	OR^a^	(95% CI)^b^	*P* value^c^
Age at enrolment (years)	< 20	1	…	…				1	…	…	** **	** **	** **
	≥ 20	0.42	(0.19–0.91)	**0.029**			** **	0.45	(0.24–0.84)	**0.012**	** **	** **	** **
Gravidity	Primigravidae	1	…	…	1	…	…	1	…	…	1	…	…
	≥ 2 pregnancies	0.31	(0.14–0.68)	**0.004**	0.24	(0.10–0.58)	**0.001**	0.36	(0.19–0.68)	**0.002**	0.31	(0.16–0.60)	**0.001**
Gestational age at enrolment	1st trimester	1	…	…				1	…	…			** **
	2^nd^/3^rd^ trimester	1.01	(0.29–3.55)	0.993				1.25	(0.43–3.67)	0.685			** **
Level of education (n = 475)	Illiterate/ Primary school	1	…	…				1	…	…			** **
	Secondary school/ Higher education	0.85	(0.37–1.94)	0.697				0.67	(0.32–1.40)	0.284			** **
Place of residence (n = 488)	Manaus area	1	…	…				1	…	…			** **
	Outside Manaus area	0.65	(0.24–1.77)	0.402				0.75	(0.36–1.56)	0.444			** **
Total number of antenatal visits (throughout pregnancy)	< 4	1	…	…				1	…	…			
	≥ 4	0.70	(0.30–1.68)	0.429			** **	0.85	(0.43–1.68)	0.636			
Clinical malaria episodes during pregnancy (any *Plasmodium* species)	1	1	…	…				1	…	…			
	≥ 2	0.60	(0.28–1.30)	0.194				0.78	(0.42–1.43)	0.416			
*Plasmodium* species at first episode in the pregnancy	*P*. *vivax*	…	…	…				1	…	…			
	*P*. *falciparum*	…	…	…				1.39	(0.66–2.93)	0.390			
	*P*. *falciparum/vivax*	…	…	…				1.83	(0.21–15.65)	0.582			
Anemia at enrolment	No anemia	1	…	…	1	…	…	1	…	…	1	…	…
	Overall anemia	2.23	(0.86–5.78)	0.099	2.56	(0.97–6.76)	0.059	1.93	(0.94–3.98)	0.073	2.14	(1.03–4.45)	**0.043**

^a^Odds Ratio.

^b^95% confidence interval.

^c^Chi-squared test.

In the group of women with malaria due to any *Plasmodium* species, the risk of LBW was highest among primigravidae and in those women with anaemia at the time of enrolment (see Table 4). There was a trend for an increased risk of low birth weight among the youngest mothers, but this was not confirmed in the multivariate analysis.

## Discussion

This study describes results on poor pregnancy outcomes among the largest series to date of pregnant women with clinical malaria in the American region. Among pregnant women with malaria, babies born to primigravidae and those who reported lesser attendance to ANC had an increased risk of suffering from low birth weight and prematurity. Also, the results serves as a paradigm to illustrate the pattern in the malaria transmission dynamics over the last years in the Amazon area where *Plasmodium vivax* has become the predominant species, and show that *P*. *vivax* malaria might be responsible for negative consequences of malaria during pregnancy in the region.

In this study, primigravidae with malaria were found to be at a higher risk for LBW and preterm delivery compared to multigravidae. This is consistent with previous data from other vivax malaria endemic areas [9] and from *P*. *falciparum* stable transmission areas as in sub-Saharan Africa [5]. Moreover, most women (82%) diagnosed with malaria in this series were young (less than 30 years old) and nearly a third of study women were adolescents (less than 19 years old). This observation may be explained by the fact that the rate of adolescent pregnancies in Northern Brazil is the highest rate in the country, and that young women are at a greater risk for malaria infection [5, 31]. The latter is well documented for *P*. *falciparum* endemic areas, where young mothers are more likely to suffer from malaria infection and its adverse effects than older ones, but few studies have reported this for *P*. *vivax* [5, 17, 32].

Most women in the study (73%) attended to ANC at least four times during pregnancy, according to World Health Organization recommendations, which is consistent with the antenatal care coverage data reported in the area. Nevertheless more than a quarter of them had less than the recommended number of visits, and this was exactly the group who showed the greater risk of suffering the negative effects of malaria during pregnancy. The reasons behind poor utilization of antenatal care services in this area are still unknown. The health seeking behaviour, barriers to access and use of health services, or other determinants associated with economic, environmental, or cultural factors could explain poor compliance.

The total of malaria episodes included in this study (n = 1016) represented 67.9% out of the 1496 malaria episodes in pregnant women reported in the Manaus area during the study period [4]. In this study, after the first malaria episode, every single study woman were encouraged to attend several follow up visits that included active detection of *Plasmodium* infection. This screening was performed monthly until delivery allowing the detection of any subsequent infection, regardless the presence of symptoms. Secondly, the study women followed weekly chemoprophylaxis with chloroquine until delivery. The positive effect of this intervention on poor pregnancy outcomes, such as LBW and maternal anaemia, and prevention of relapses, has been demonstrated in several studies, yet none of the studies included pregnant women from Latin America [33, 34]. It should be noted that, among study women, this prompt detection and treatment of any malaria infection together with the prophylactic regimen may have had an effect in decreasing the risk of negative outcomes, as these interventions would have curtailed infections at an early stage, differently to what might occur in the group of pregnant women that did not participate in the study [9].

Interestingly, this study reveals that more than one third of the study women (36%) had more than one malaria episode during the same pregnancy despite the weekly chemoprophylaxis with chloroquine. The contraindication during pregnancy of radical cure with primaquine due to safety issues, putting women at a higher risk of subsequent episodes, as well as the chloroquine resistance reported in the area may explain this finding. To date, no study has shown if pregnant women have an increased risk of relapses compared to non-pregnant populations. In a Cochrane meta-analysis aimed to compare alternative primaquine regimens for preventing relapses in people with *P*. *vivax* malaria, the frequency of relapses in control groups (no primaquine) was 20%, but this review did not include data from pregnant women or from Latin America [35]. Although the design of the current study does not allow showing if this risk is higher among pregnant women, it reminds that these women are more vulnerable to *P*. *vivax* malaria since radical treatment of *P*. *vivax* infections with primaquine is contraindicated during pregnancy.

The prevalence of maternal anaemia found in this study (59%) was considerably higher compared with the prevalence of anaemia found among women attending routine ANC from two studies conducted in the area in women with similar characteristics attending the routine antenatal care, being at 26% in 2008 [36] and at 30%, in 2009–2010 [A. Bardají, personal communication]. This finding supports that *P*. *vivax* malaria can be a considerable cause of maternal morbidity, which is consistent with previous studies from this and other *P*. *vivax* endemic areas [9–11, 16].

The change in the distribution of species observed over the study period, showing that *P*. *vivax* gradually prevailed over *P*. *falciparum*, are aligned with the change in the pattern observed in general population in this region [22]. This also includes a reduction in the malaria incidence in the region. The treatment guidelines for *P*. *vivax* malaria have not changed in recent years in Brazil [22]. Thus, these changes might be explained by the significant expansion and strengthening of the diagnosis network towards the peripheral areas of the Manaus city, which allowed to improve the case management, together with the introduction of Artemisinin-based Combination Therapy for the treatment of *P*. *falciparum* malaria in 2006.

The prevalence of LBW seen was similar to that observed in the general population (8.1%), which is similar in terms of demographic and social characteristics to that in our study [20]. LBW was primarily associated with premature delivery, and with foetal growth restriction to a lesser extent; however the prevalence of premature delivery showed more than two-fold increase with respect to the general population of Manaus (14.8% vs. 6.6%) [20]. One hypothesis that might explain the higher frequency of prematurity but not of LBW would be that in the Brazilian Amazon, an area of unstable malaria transmission where women are not immune and respond clinically to a *Plasmodium* sp. infection, each episode of malaria exposes the mother to the malaria paroxysm, which may be associated with increased uterine activity and thus triggering a premature labour [17]. The studies of the mechanisms of *P*. *vivax* paroxysm have shown all the cellular and noncellular mediators involved [37]. One of them is the pyrogenic cytokine tumor necrosis factor (TNF)-α, which also has been shown to be increased in *P*. *falciparum* placental malaria, and to be embryotoxic, cause necrosis of the implanted embryo, and increase the risk of uterine contraction resulting in foetal expulsion [38].

In this study, as shown in Fig 2, most LBW babies were appropriate for gestational age (between 10^th^ and 90^th^ percentiles), being only few of them (9%) below the 10^th^ percentile (IUGR). This is in smaller than IUGR rates reported in other Brazilian studies (13%) [39]. Hence, it seems that premature birth was the main responsible for LBW, and that the contribution of malaria infection to the impairment of foetal growth was less evident. This suggest that, in areas of unstable malaria transmission where *P*. *vivax* predominates, prematurity appears to be among the main detrimental outcomes of malaria in pregnancy, differently to what occurs in *P*. *falciparum* areas where transmission is stable and LBW is the main outcome associated with malaria infection in the pregnant woman followed by pre-term birth.

There are studies that indicate that male and female foetuses behave differently upon the same adverse maternal environment, resulting in different foetal and neonatal morbidity and mortality outcomes [30]. Studies showed that malaria in pregnancy was associated with decreased mean birth weight of female newborns, but not of male newborns, and that female fetus had a higher risk of IUGR compare to male [29, 40]. In contrast, in our cohort of newborns, sex specific differences in fetal growth (increased risk of IUGR) between female and male newborns were seen (9.7% vs.8.1%), but these were not statistically different.

A limitation of this study was that birth weight was obtained from the SINASC system, however, among a subsample of newborns, there was a high correlation between this weight and that collected from the newborn card by study staff. Gestation age was established by ultrasound in 61.2% (295/482) and it is known that last menstrual period is subject to reporting bias. Other poor pregnancy outcomes in this study population such as miscarriage and stillbirth are reported elsewhere [17]. Also, it should be noted that the design of this study does not permit to estimate the impact of *P*. *vivax* malaria on pregnancy outcomes, as a comparison group of non-infected women were not enrolled.

In conclusion, this study shows that *P*. *vivax* is the prevailing species among malaria cases in pregnant population in the region and shows that *P*. *vivax* malaria in pregnancy may represent detrimental consequences for the health of the mother and their offsprings particularly for specific groups such as primigravidae, adolescents, and those with lower attendance to antenatal services with *P*. *vivax* clinical malaria during pregnancy. These groups deserve special attention in the design and deployment of preventive and curative strategies for malaria control during pregnancy.
